# Genome sequencing and analysis of black flounder (*Paralichthys orbignyanus*) reveals new insights into Pleuronectiformes genomic size and structure

**DOI:** 10.1186/s12864-024-10081-z

**Published:** 2024-03-20

**Authors:** Fernando Villarreal, Germán F. Burguener, Ezequiel J. Sosa, Nicolas Stocchi, Gustavo M. Somoza, Adrián G. Turjanski, Andrés Blanco, Jordi Viñas, Alejandro S. Mechaly

**Affiliations:** 1 Facultad de Ciencias Exactas y Naturales, Instituto de Investigaciones Biológicas (IIB-CONICET-UNMdP), Universidad Nacional de Mar del Plata, Mar del Plata, Argentina; 2 Plataforma de Bioinformática Argentina, Facultad de Ciencias Exactas y Naturales, Instituto de Cálculo, UBA, Pabellón 2, Ciudad Universitaria, Buenos Aires, Argentina; 3 Instituto de Química Biológica de la Facultad de Ciencias Exactas y Naturales (IQUIBICEN) CONICET, Ciudad Universitaria, Buenos Aires, Argentina; 4 Instituto Tecnológico de Chascomús (CONICET-UNSAM), Chascomús, Buenos Aires, Argentina; 5 Escuela de Bio y Nanotecnologías (UNSAM), Buenos Aires, Argentina; 6 Departamento de Química Biológica, Facultad de Ciencias Exactas y Naturales, Universidad de Buenos Aires, Buenos Aires, Argentina; 7 Facultade de Veterinaria, Universidade de Santiago de Compostela, Santiago de Compostela, Lugo, Spain; 8 Departamento de Zoología, Genética y Antropología Física, Facultad de Veterinaria, Campus Terra, Universidade de Santiago de Compostela, Lugo, Spain; 9 Laboratori d’Ictiologia Genètica, Departament de Biologia, Universitat de Girona, Maria Aurèlia Campmany, 40 Girona, Spain; 10 Instituto de Investigaciones en Biodiversidad y Biotecnología (INBIOTEC-CONICET), Mar del Plata, Argentina; 11 Fundación Para Investigaciones Biológicas Aplicadas (FIBA), Mar del Plata, Argentina

**Keywords:** Pleuronectiformes, *Paralichthys orbignyanus*, Black flounder, Genome, Intron size, Ensembl, Evolution

## Abstract

Black flounder (*Paralichthys orbignyanus*, Pleuronectiformes) is a commercially significant marine fish with promising aquaculture potential in Argentina. Despite extensive studies on Black flounder aquaculture, its limited genetic information available hampers the crucial role genetics plays in the development of this activity. In this study, we first employed Illumina sequencing technology to sequence the entire genome of Black flounder. Utilizing two independent libraries—one from a female and another from a male—with 150 bp paired-end reads, a mean insert length of 350 bp, and over 35 X-fold coverage, we achieved assemblies resulting in a genome size of ~ 538 Mbp. Analysis of the assemblies revealed that more than 98% of the core genes were present, with more than 78% of them having more than 50% coverage. This indicates a somehow complete and accurate genome at the coding sequence level. This genome contains 25,231 protein-coding genes, 445 tRNAs, 3 rRNAs, and more than 1,500 non-coding RNAs of other types. Black flounder, along with pufferfishes, seahorses, pipefishes, and anabantid fish, displays a smaller genome compared to most other teleost groups. In vertebrates, the number of transposable elements (TEs) is often correlated with genome size. However, it remains unclear whether the sizes of introns and exons also play a role in determining genome size. Hence, to elucidate the potential factors contributing to this reduced genome size, we conducted a comparative genomic analysis between Black flounder and other teleost orders to determine if the small genomic size could be explained by repetitive elements or gene features, including the whole genome genes and introns sizes. We show that the smaller genome size of flounders can be attributed to several factors, including changes in the number of repetitive elements, and decreased gene size, particularly due to lower amount of very large and small introns. Thus, these components appear to be involved in the genome reduction in Black flounder. Despite these insights, the full implications and potential benefits of genome reduction in Black flounder for reproduction and aquaculture remain incompletely understood, necessitating further research.

**Supplementary Information:**

The online version contains supplementary material available at 10.1186/s12864-024-10081-z.

## Introduction

Pleuronectiforms represent a fish order of significant biological interest due to their adaptations to demersal life and their remarkable metamorphosis, transitioning from bilateral pelagic larval symmetry to flatfish symmetry in adulthood [1, 2]. This order comprises of a total of 772 species distributed across 14 families and 129 genera [3]. Some of these species are economically important for fisheries and aquaculture [4]. One particularly relevant genus for aquaculture within this order is *Paralichthys*. For example, the Japanese flounder (*Paralichthys olivaceus*) has a well-established aquaculture industry in Asia [5]. In Latin America, other *Paralichthys* species hold potential importance for fisheries and aquaculture. Among them, the Black flounder (*Paralichthys orbignyanus*) in Brazil, Argentina, and Uruguay [6] and the Chilean flounder (*Paralichthys adspersus*), endemic to Peru and Chile, are species of significant regional interest.

The Black flounder inhabits shallow estuaries and coastal waters from Rio de Janeiro [6] to the Gulf of San Matías in northern Patagonia [7]. Despite numerous zootechnical studies conducted over the last two decades to explore its aquaculture potential [8–15], there remains a significant knowledge gap regarding the genetic resources of this species. For example, only 12 protein sequences are deposited in the GenBank® sequence database which is particularly remarkable given that genetics and genomics play a crucial role in the development of the aquaculture and fisheries industry [2, 16].

Genetic information is essential for aquaculture and fisheries management [17], supporting selective breeding for improved traits, enhanced disease resistance, genetic diversity maintenance, and sustainable practices. Furthermore, it facilitates product traceability, species conservation and adaptation to environmental changes, all vital for responsible and effective management of fish populations and ecosystems. The market demand, high nutritional value, and aquaculture potential of the Black flounder in South America position it as a promising candidate for marine farming, although several challenges are yet to be addressed. One of the initial hurdles is its slow growth rate, contrasting with the rapid growth observed in nature [18]. Additionally, resolving the nutrition aspect requires the development of economically and environmentally sustainable fish food for this species. Moreover, the development of Black flounder farming in South America, especially in Argentina, demands market analysis and bioeconomic models to formulate sustainability strategies for this product. Considering these factors, along with the study of sex determination and differentiation, sex ratio, nutrition, reproduction, genetic population structure, and other essential biological traits, obtaining a reference genome is a fundamental step in investigating these processes and mechanisms. Undoubtedly, it would significantly contribute to the promotion of Black flounder aquaculture in South America.

Over the last decade, next-generation sequencing (NGS) technologies have gained acceptance and played a critical role in obtaining whole genome sequences from various non-model fish species. According to the *Ensembl* Genome Browser (https://www.ensembl.org), hundreds of fish species have whole genome sequences (WGS) are available to date, with the number continuously increasing. Among Pleuronectiformes, thirteen species have their genomes sequenced, including Tongue sole (*Cynoglossus semilaevis*) [19], Turbot (*Scophthalmus maximus*) [20], Senegalese sole (*Solea senegalensis*) [21], Japanese flounder (*Paralichthys olivaceus*) [22], Hogchoker (*Trinectes maculatus)*, Pelican flounder (*Chascanopsetta lugubris)*, Oriental sole (*Brachirus orientalis)*, Bloch's tongue sole (*Paraplagusia blochii)*, New Zealand turbot (*Colistium nudipinnis)*, Ocellated flounder (*Pseudorhombus dupliocellatus)*, Starry flounder (*Platichthys stellatus)*, and Indian halibut *Psettodes erumei*) [1]. Furthermore, recent studies have compiled the genomes at the chromosome level for Turbot [23] and Spotted halibut (*Verasper variegatus*) [24], or analyzed the origin of the specialized body structure of flatfishes by sequencing the genomes of 11 flatfish species representing 9 of the 14 Pleuronectiformes families [1]. In this context, the sequencing and annotation of the Black flounder genome would expand the knowledge of this species, enabling the use of alternative genomic methods to improve fish breeding and conduct comparative and evolutionary studies with other flatfish species.

Comparative genomics has revealed striking differences in genome size, even in closely related species, with considerable variation in teleost, not only in genome size but also in chromosome number [25–27]. Previous studies have demonstrated that Pleuronectiformes, along with pufferfishes, seahorses, pipefishes, and Anabantiformes exhibit the smallest genome among teleost groups [28, 29]. Some researchers have associated the reduction of the genome size in amniotes with a decrease in intron size [30]. Alternatively, genome size has also been correlated with the frequency and size of repetitive elements [25]. Notably, Pleuronectiform species contain less than 9.0% repetitive elements [31], in stark contrast to species like the sea lamprey (*Petromyzon marinus*), coelacanth (*Latimeria chalumnae*), and salmon (*Salmo salar*) genomes, which exhibit approximately 60% repetitive elements [32–34]. However, the reasons and implications of this observed reduction in the genomes of these fish remain unclear. Generally, variations in genome size are explained by the interplay of different genome-level mechanisms that either lead to genome expansion (via duplication, transposable elements, and polyploidy) or genome reduction (involving deletions and DNA repair mechanisms) [35].

The primary objective of this study was to sequence and characterize of the whole genome of Black flounder, thereby providing a crucial genomic resource for further research on this species. Additionally, we conducted a comparative genome analysis to investigate whether Black flounder, among other Pleuronectiformes, indeed possesses a smaller genome size compared to other teleost species. This analysis involved comparing the size of genes and other gene features (exons and introns), as well as examining transposable elements and other repetitive elements in different Pleuronectiformes in relation to the genomes of other teleost species.

## Material and methods

### Fish sampling and DNA extraction

Live adult Black flounder, *Paralichthys orbignyanus* were obtained from the Estación Experimental de Maricultura (INIDEP, Argentina). Prior to dissection, fish were anesthetized with tricaine methanesulfonate (MS-222, Sigma-Aldrich). Tissue samples were then excised from the caudal fin of live specimens and preserved in 96% ethanol. Immediately before dissection, the fins were treated with iodine, and the fish were immediately transferred to a recovery tank with oxygen. The fish were then observed for an appropriate period to ensure that they fully recovered from anesthesia. No mortalities were recorded either during sampling or in the days that followed. Genomic DNA was extracted from one adult female and one male. Samples were lysed in 300 μl SSTNE extraction buffer [36] with SDS (0.1%) and 5 μl proteinase K (20 mg/ml) for 3 h at 55 °C. After 20 min at 70 °C, samples were treated for RNA digestion with 7.5 μl RNAse (10 mg/ml) for 1 h at 37 °C. Total DNA was purified with cold absolute ethanol (1 ml) after protein precipitation with 5 M NaCl. DNA quality (high molecular weight > 20 kb) was first assessed on agarose gels and DNA quantity was measured using an ND-1000 spectrophotometer (NanoDrop® Technologies Inc). Finally, DNA concentration was accurately measured using a Qubit fluorometer (Life Technologies). Fish were handled in accordance with international animal welfare regulations.

### Genome sequencing and genome assemblies

All samples were adjusted to 300 ng/µL and fragmented by ultrasonication, aiming for a fragment size of 300 base pairs (bp). Barcoded libraries were prepared using Wafergen's PrepX ILM DNA Library Kit and beads size selected before being pooled and sequenced at 150 bp at the paired end on HiSeq-4000. All procedures followed the manufacturer's instructions. We performed multiple QC sets during library preparation using 300 ng of human gDNA samples as internal control and all QC passed for this project.

Two Illumina paired-end libraries were constructed (from one female and one male) and sequenced with a read length of 150 bp and an average fragment size of ~ 350 bp. Reads were first filtered using Trimmomatic [37] and PrinSeq [38] to remove adapters and low-quality reads, and then sequencing errors were corrected using Quake [39]. The libraries were assembled individually using SOAPdenovo 2.04 [40, 41] with a kmer size of 29, gap filled with GapCloser [42] and finally scaffolded with SSPACE3 [43] with default parameters.

### Genome annotation

In order to comprehensively annotate the genomic elements of the fish, we initiated the annotation process by applying Repeatmasker [44, 45] to mask repetitive elements, utilizing the Repbase [46] and DFam [47] databases. Subsequently, the masked scaffolds underwent a multi-step annotation strategy. Transfer RNA (t-RNA), ribosomal RNA (r-RNA), and non-coding RNA (nc-RNA) sequences were identified using tRNAScan-SE [48], RNAmmer [49], and Infernal [50] in conjunction with the RFAM database [51].

Next, gene prediction was carried out using GeneId [52] and Exonerate [53], both trained with information from Uniprot/Swissprot [54] and the genome of the Japanese flounder (*Paralichthys olivaceus*) [22]. To further enhance the completeness of our predictions, all predicted proteins underwent annotation using InterProScan [55, 56]. which added InterPro [57], gene ontology, PFAM [58] among others. This comprehensive annotation strategy ensured a robust identification and classification of genomic elements within the studied fish genome.

### Gene family clustering and validation

To find orthologous proteins in other fish species, we downloaded the genome data of *D. rerio* [59], *C. semilaevis* [19], *S. maximus* [60] and *P. olivaceus* [22] from the Zebrafish Genome Project and *Ensembl*. All protein sequences were aligned using BLASTP [61] with an E value cutoff lower than 1e − 5. BLASTP results were parsed and imported into a MySQL database. Tables within were created by OrthoMCL [62] to identify homologues with thresholds of percentMatchCutoff = 50 and evalueExponentCutoff = 1e − 6. To evaluate our annotation, we used Benchmarking Universal Single-Copy Orthologs (BUSCO, v3.0.237) [63, 64] to assess the assembled genome sequences. We used BUSCO with single copy orthologues from actinopterygii_odb10 to assess the completeness of the genome assembly and annotation. GO terms lists were simplified by means of semantic similarity using REVIGO [65], with a cutoff value *C* = 0.5 and normalized Resnik similarity measure.

### Identification and comparison of Repetitive elements (REs)

We quantified several Repetitive elements (REs), among them Transposable Elements (TEs), Long or Short Interspersed Nuclear Elements (LINEs and SINEs, respectively) and Long Terminal Repeats (LTRs) for each fish genome. To do so, we applied a homology-based approach [44] using RepeatMasker with Actinopterygii species repeats and default parameters, which we ensure to only annotate and mask repeats less than 20% diverged to its consensus sequence (setting -div option to 20), return the alignments in the orientation of the repeat consensus sequences (-inv option), and set the percentage of GC level to 45% and use this value to choose the optimal matrix for the algorithm (setting -GC option to 45). Relationships between genome size and TEs percentage in the genome were plotted using ggplot2 R package, and correlations were calculated by Spearman method.

### Sex determination genes search in the Black flounder

The search for sex determination genes in flounder is critical to understanding the mechanisms controlling sex determination, which may have significant implications for controlling sex ratios in aquaculture and improving breeding programs. The BLAST 2.2.29 algorithm was used to retrieve the genomic sequences of SRY-Box Transcription Factor 2 (*Sox2*), follicle stimulating hormone receptor (*fshr*), bone morphogenetic protein receptor type-1B (*bmpr1ba*), forkhead box L2 (*Foxl2),* doublesex, mab-3 related transcription factor 1 (*Dmrt1),* gonadal soma-derived factor (*gsdf*), and a male-specific duplication of anti-Müllerian hormone (*amh*) from the Black flounder genome database using other Pleuronectiforms orthologous.

### Analysis of C-value in teleosts

All fish C-values (haploid nuclear DNA content in pg) were retrieved from the Animal Genome Size Database [66]. As of July 2023, the website featured information on over 1800 fish species spanning across more than 70 orders, with 48 entries specifically dedicated to Pleuronectiformes species. The data in the database was primarily obtained through wetlab techniques, such as flow cytometry and Feulgen densitometry. In cases where multiple entries existed for the same species, the average Cvalue was computed. When necessary, C-values were estimated based on genome size in base pairs, utilizing the conversion factor of 1 pg = 978 Mbp [67]. Boxplots were generated using Plotly (Plotly Technologies Inc. Collaborative data science. Montréal, QC). Because most data sets did not show a normal distribution (according to the Shapiro–Wilk test), C-value data were analyzed with the Mann–Whitney test, comparing Pleuronectiformes with each order using JASP (version 0.16, JASP Team, 2021).

### Gene features size analysis at whole genome scale in teleost orders

Complete genome annotations were retrieved from ENSEMBL in gff3 format. We selected species in orders with diverse phylogenetic relationship to Pleuronectiformes. A total of twenty seven species representatives from ten fish orders were used: Anabantiformes (*Anabas testudineus*, *Betta splendens*, *Mastacembelus armatus*), Carangiformes (*Echeneis naucrates*, *Seriola dumerili*, *Seriola lalandi dorsalis*), Cichliformes (*Astatotilapia calliptera*, *Oreochromis niloticus*, *Pundamilia nyererei*), Cypriniformes (*Cyprinus carpio*, *Danio rerio*, *Sinocyclocheilus rhinocerous*), Cyprinodontiformes (*Fundulus heteroclitus*, *Kryptolebias marmoratus*, *Poecilia formosa*), Perciformes (*Cyclopterus lumpus*, *Gasterosteus aculeatus*, *Sander lucioperca*), Pleuronectiformes (*Cynoglossus semilaevis*, *Paralichthys orbignyanus* female, *Scophthalmus maximus*), Salmoniformes (*Hucho hucho*, *Oncorhynchus mykiss*, *Salmo trutta*), Labriformes (*Labrus bergylta*) and Tetraodontiformes (*Takifugu rubripes*, *Tetraodon nigroviridis*)*.* We chose to use the assembly from Black flounder’s female because of the higher coverage achieved. See Supplementary Table S1 for additional details. We retrieved the size of each gene model (genomic *locus*) by calculating the difference between the final and initial base coordinates. For each model at a given locus, we calculated the number and size of each exon. Finally, intron sizes were inferred by calculating the difference between the first base coordinate of an exon and the last base coordinate of the preceding exon.

The size distribution of exons and introns at the genome level were plotted using the kernel density estimation of the Seaborn package (parameters: gaussian kernel, band width 0.1 for exons and 0.01 for introns, and grid size of 1000 for exons and 5000 for introns). Boxplots for the size distributions were plotted using Plotly.

### Results genome annotation

After filtering, the remaining reads accounted for a coverage of more than 35 X-fold on each genome and were retained for assembly (Table 1).

**Table 1:** Genome coverage for each library. Genome sequencing (post-filtering)

Sample	Library	Read Length (bp)	Insert Size (bp)	Coverage
Female	*pair-end*	150	~ 350	38.7X
Male	*pair-end*	150	~ 350	35.8X

The final assemblies were around 524–538 Mb, with the female being the less fragmented (Table 2).

**Table 2:** Black flounder assembly statistics

Assembly	Female	Male
# Contigs (≥ 1000 bp)	78,084	75,834
Scaffold N50 Size (bp)	11,125	11,694
Longest Scaffold (bp)	194,365	185,663
Total Scaffold Length (bp)	538,587,025	540,288,577
GC Content (%)	41.39	41.38
%N	0.13	0.09

To verify annotation completeness, we checked for BUSCO orthologues in Actinopterygii DB, where 3459 (3252 complete and 198 fragmented from a total of3652 entries (94,7%) were identified in the combined assemblies (for more information see Table 3).

**Table 3:** Gene product annotations results, GeneId, Exonerate, Infernal (with RFAM, DB, tRNAScan-SE and RNAmmer)

	Female	Male
Proteins	25,262	25,231
KOG	3541	3670
Uncharacterized	~ 4%	~ 4%
tRNAs (all types)	509	524
rRNAs	11	9
snRNAs	49	57
Others ncRNAs	971	929

### Transposable elements (TEs) identification and quantification

**Fig. 1:**
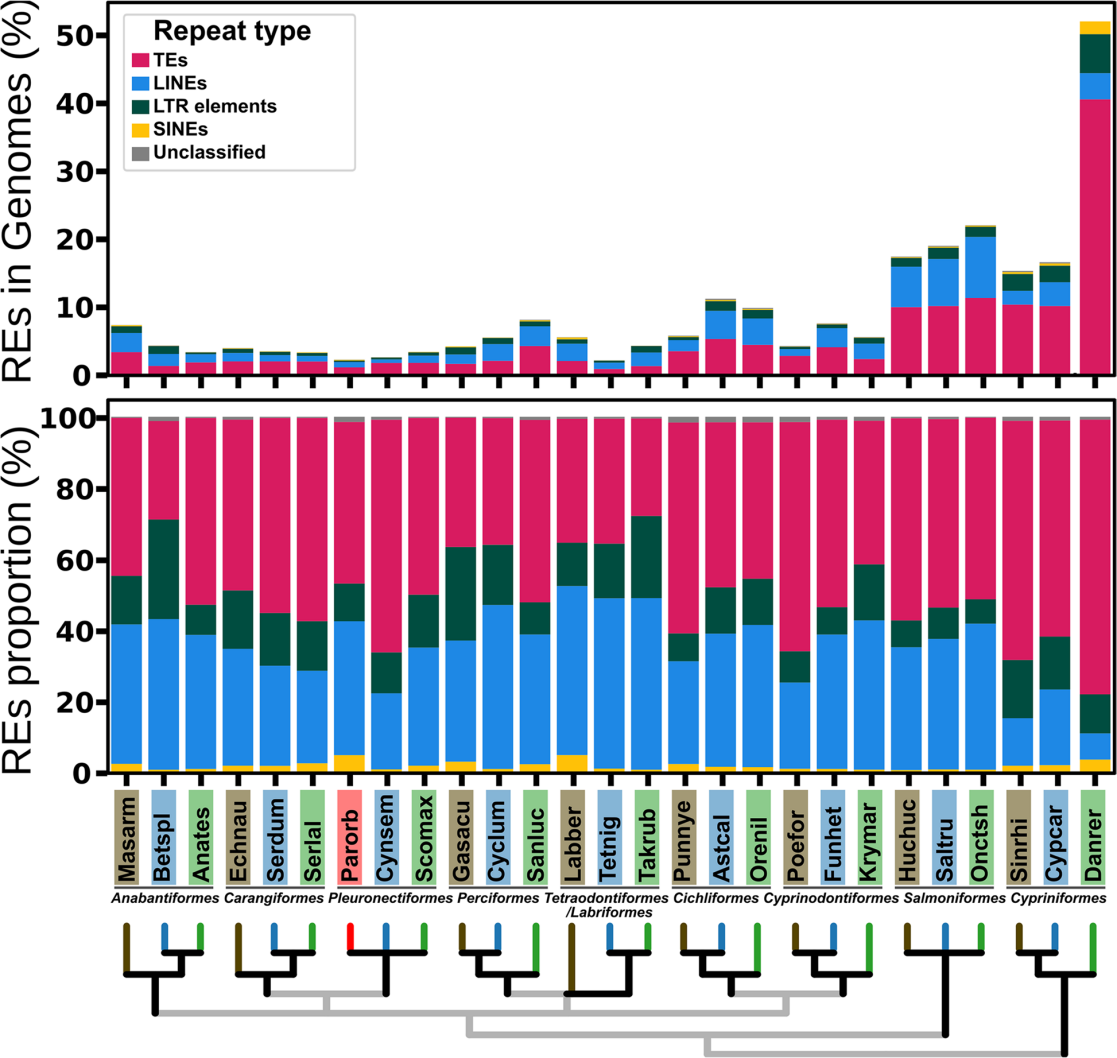
Contents of repetitive elements in fish genomes. Several repetitive elements (REs), such as transposable elements (TEs), long and short interspersed nuclear elements (LINEs and SINEs, respectively) and long terminal repeats (LTRs) are shown both as total percentage in genome (A) or percentage relative to repetitive elements content (B). A taxonomy tree for the species analyzed is shown below. Species: Anabantiformes (Anates: *Anabas testudineus*, Betspl: *Betta splendens*, Masarm: *Mastacembelus armatus*), Carangiformes (Echneu: *Echeneis naucrates*, Serdum: *Seriola dumerili*, Serlal: *Seriola lalandi dorsalis*), Cichliformes (Astcal: *Astatotilapia calliptera*, Orenil: *Oreochromis niloticus*, Punnye: *Pundamilia nyererei*), Cypriniformes (Cypcar: *Cyprinus carpio*, Danrer: *Danio rerio*, Sinrhi: *Sinocyclocheilus rhinocerous*), Cyprinodontiformes (Funhet: *Fundulus heteroclitus*, Krymar: *Kryptolebias marmoratus*, Poefor: *Poecilia formosa*), Perciformes (Cyclum: *Cyclopterus lumpus*, Gasacu: *Gasterosteus aculeatus*, Sanluc: *Sander lucioperca*), Pleuronectiformes (Cynsem: *Cynoglossus semilaevis*, Parorb: *Paralichthys orbignyanus*, Scomax: *Scophthalmus maximus*), Salmoniformes (Huchuc: *Hucho hucho*, Onctsh: *Oncorhynchus tshawytscha*, Saltru: *Salmo trutta*), Labriformes (Labber: *Labrus bergylta*) and Tetraodontiformes (Takrub: *Takifugu rubripes*, Tetnig: *Tetraodon nigroviridis*)

**Fig. 2:**
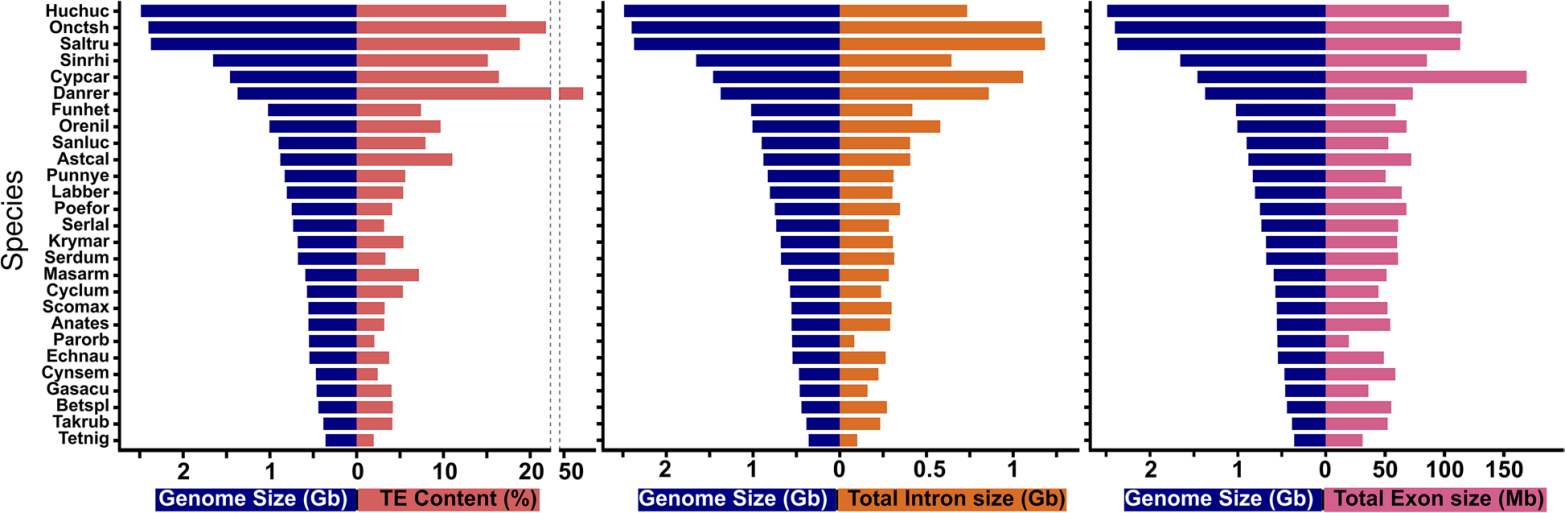
Correlation between genome size (Gb) and TE content (%), Total intron size and Total exon size of fish species. Species analyzed are represented by the sixletter code as described in Fig. 1

RepeatMasker showed large differences in the frequency of several repetitive elements (REs) between fish species. Figure 1 compares percentage and proportion of transposable elements (TEs) and other REs (LINEs, SINEs and LTRs) among different fish genomes. In general, lineages with larger genome sizes had a higher frequency of REs (Fig. 1). In the same line, small genomes such as *T. nigroviridis* and Pleuronectiformes (especially *P. orbignyanus*) presented a very low frequency of REs in general. Among the Pleuronectiformes, the genome of *P. orbignyanus* has a lower proportion of DNA transposons and a higher proportion of long (LINEs) and short (SINEs) interspersed nuclear elements. We also studied the relationship between genome size with different genome elements, showing strong correlation with TEs percentage (*r* = 0.847; *p* < 0.001), total intron size (*r* = 0.939; *p* < 0.001) and total exon size (*r* = 0.807; *p* < 0.001) (Fig. 2).

### Gene family clustering

**Fig. 3:**
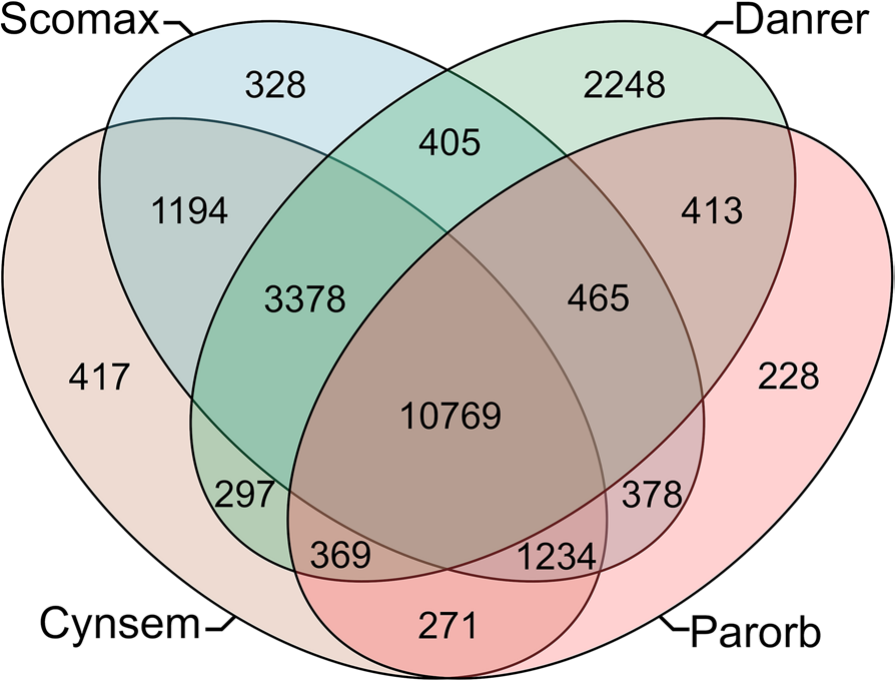
Venn diagram showing orthology in the four flatfish species (*C. semilaevis*, *S. maximus*, *P. olivaceus,* and* P*. *orbignyanus* and zebrafish. Protein Orthologs were calculated using OrthoMCL

Gene families were clustered according to their similar structures into three different flatfish families: Scophthalmidae (*S. maximus*), Paralichthyidae (*P. orbignyanus* and *P. olivaceus*), Cynoglossidae (*C. semilaevis*), and *D. rerio* using OrthoMCL. A total of 10,769 putative specific genes among the five species and 1234 putative specific gene families were identified among the four flatfish species included in the analysis (Fig. 3). Among the GO terms describing the gene families shared by the flatfishes, we identified key biological features associated to flatfish’s evolution (Supplementary Table S2). In this line, we identified GO terms shared by all flatfishes that have been recently associated to genes under positive selection or rapidly evolving [1], corresponding to biological process classes such as phosphatidylinositol dephosphorylation (GO: 0046856) or protein transport (GO:0015031). Also, a number of features involved in adaptation to benthic lifestyle are also represented in the GO terms shared by all flatfishes, such as adaptation to lower light intensity, asymmetric pigmentation and body plan, and muscle development (Supplementary Table S2), which agrees with previous observations [1, 22].

### Black flounder sex determination genes

The presence of *sox2*, *fshr*, *bmpr1ba*, *foxl2, dmrt1,* and *gsdf* ORFs in the Black flounder genome database was detected using the orthologous gene of *Paralichthys olivaceus* (Japanese flounder) (PRJNA369269) as query. This suggests the presence of conserved genes related to sex determination between these two flounder species, indicating a possible shared biological functions or evolutionary relationships.

### C-value comparison between Pleuronectiformes and other fish orders

**Fig. 4:**
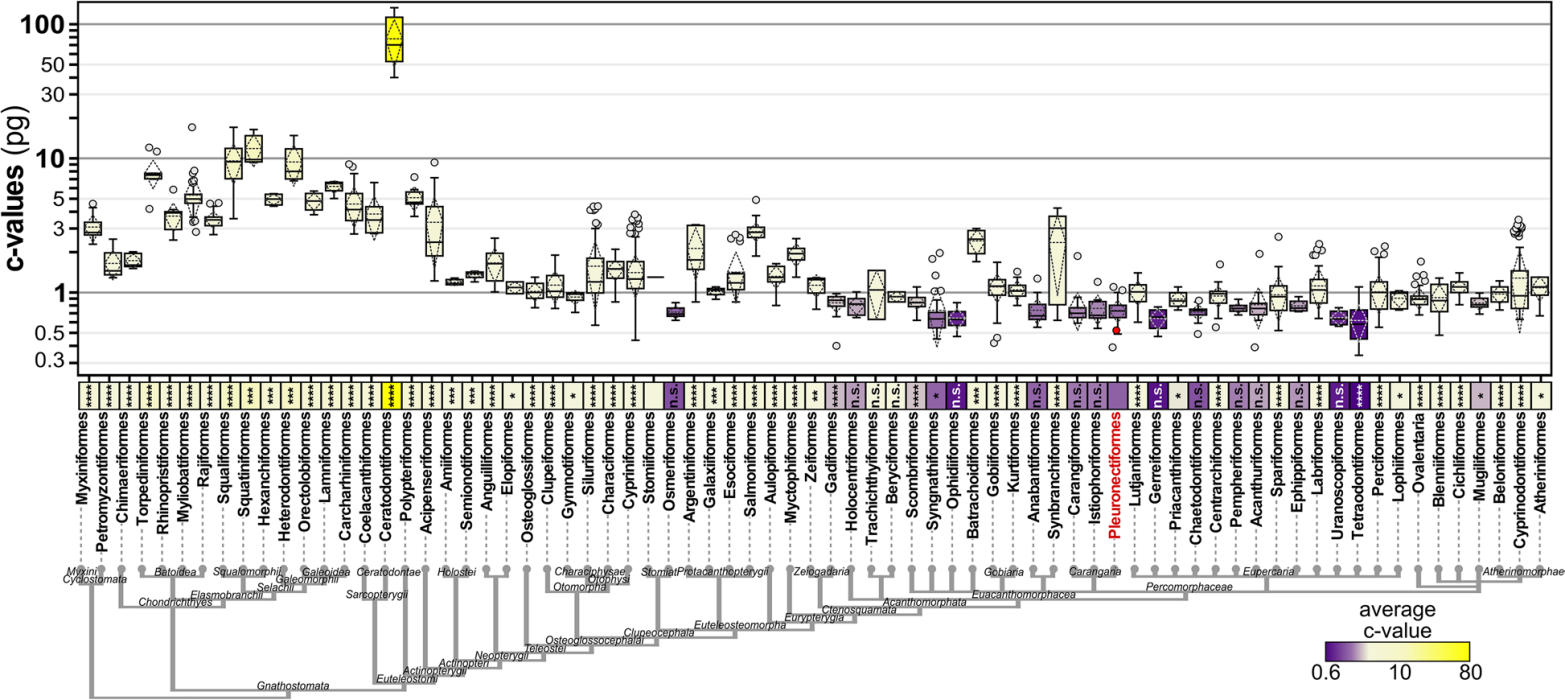
Haploid genome size across fish based on C-value. Boxplots representing C-values of 34 fish orders (top), organized by their taxonomic relationships (bottom). Y-axis represents C-values in log scale. For boxplots: horizontal bar, median; dashed lines, mean and standard deviation; circles, outliers. C-value for *P. orbignyanus*, red colored circle. C-value means represented by boxes colored from yellow (highest) to purple (lowest). Analysis of unpaired t-test of Pleuronectiformes versus each order are shown (****, *P* < 0.0001; ***, *P* < 0.001; **, *P* < 0.01; ns, not significant)

Using publicly available C-value data, we performed a comparative analysis of haploid genome sizes in fish (Fig. 4). We used data from 1504 species belonging to 73 orders (according to the NCBI taxonomy database). The estimated C-value of *P. orbignyanus* is 0.56 pg, which corresponds to a small fish genome size (5th percentile = 0.61 pg). The average C-value for Pleuronectiformes is 0.734 pg, and the only order with a significantly lower average C-value is Tetraodontiformes (0.618 pg, *P* < 0.001). Gerreiformes, Ophidiiformes, Uranoscopiformes, Osmeriformes, and Chaetodontiformes also have lower average C-value than Pleuronectiformes, but with no statistical difference. Among Pleuronectiformes, 16.3% of the species had C-values in the 5th percentile range. In addition, 51.5% of Tetraodontiformes species had C-values in the 5th percentile, consistent with the fact that this order had the lowest mean Cvalue. Only a low proportion of species in the orders represented in this 5th percentile range have a C-value less than 0.61 pg.

**Fig. 5:**
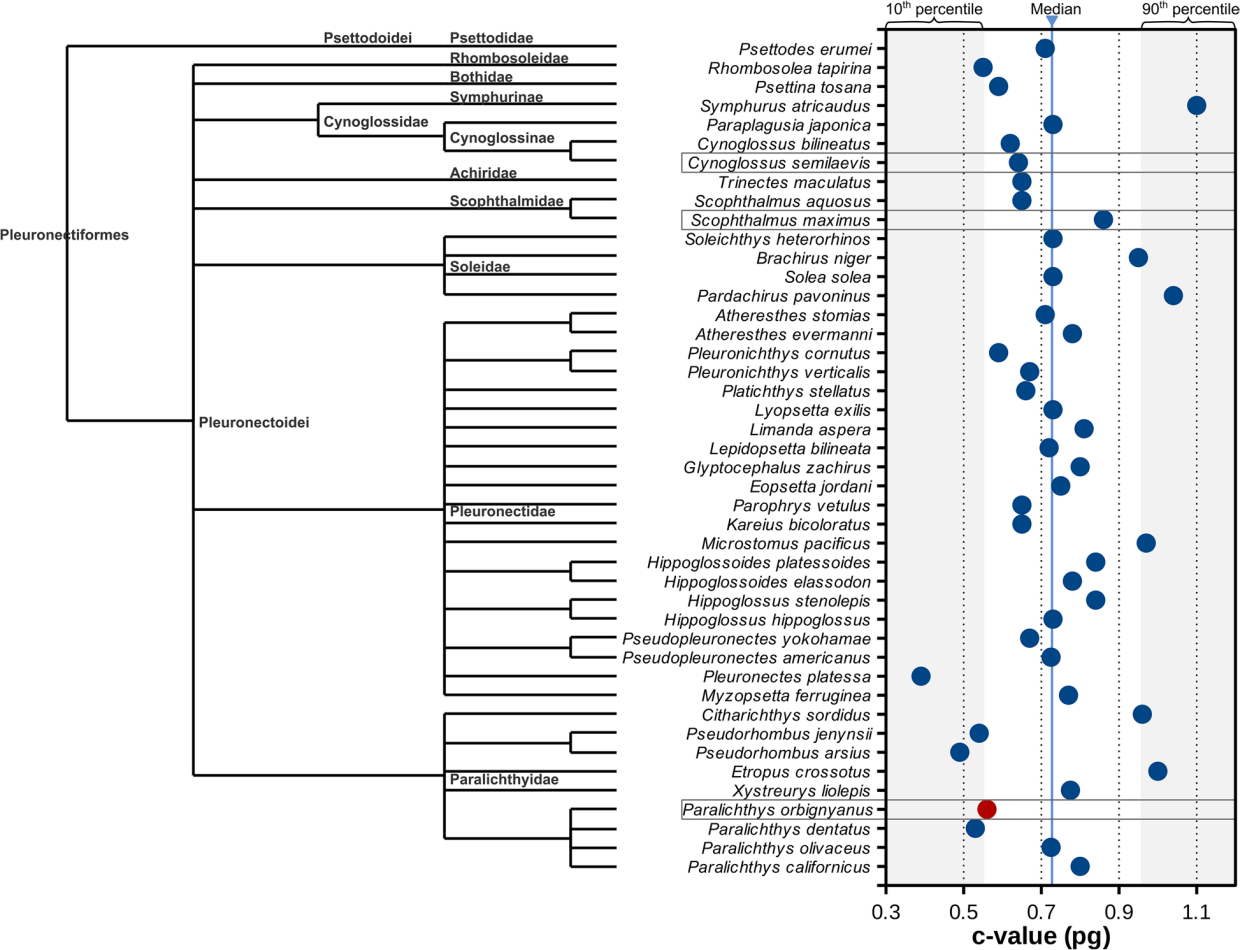
Pleuronectiformes species C-values. The median and the 10th and 90th percentile values are shown. C-value for *P. orbignyanus* shown in red. Other two species (*C. semilaevis* and *S. maximus*) with sequenced genomes are also highlighted. Taxonomy from NCBI's Common Tree for Pleuronectiformes shown on the left

We additionally analyzed the C-values of all Pleuronectiformes species (Fig. 5). The C-value of *P. orbignyanus* is one of the smallest in the order (Pleuronectiformes 10th percentile value = 0.55 pg), with three members of the family Paralichthyidae having lower C-values (*Pseudorhombus jenynsii* 0.54 pg, *Paralichthys dentatus* 0.53 pg, and *Pseudorhombus arsius* 0.49 pg). Finally, two other species from different families had smaller C-values: *Rhombosolea tapirina* 0.55 pg (Rhombosoleidae family), and *Pleuronectes platessa* 0.39 pg (Pleuronectidae family).

Taken together, these results indicate that low genome size is a trait of Pleuronectiformes, and particularly in species from Paralichthyidae, including Black flounder.

### Whole genome scale analysis of gene features size in teleost fish

#### Coding genes number and size

**Fig. 6:**
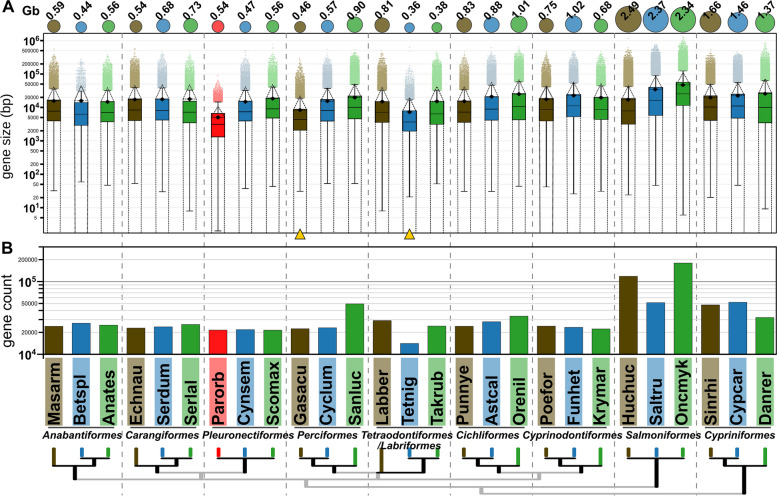
Gene size in Black flounder and other teleost fish genomes. **A** Gene size distribution of represented by boxplots (outlier values indicated by colored dots). On top, circle diameter indicates whole genome size (numeric value in Gb is also shown). Average represented by diamonds, and standard deviation in dotted lines. **B** Count of genes predicted in whole genomes. Species analyzed are represented by the sixletter code as described in Fig. 1 (Onkmyk: *Oncorhynchus mykiss*). Yellow arrows indicate species with small genome size and distribution towards smaller gene sizes (other than Black flounder)

To find other possible factors affecting the relatively small size of the *P. orbignyanus* genome, we investigated whether this might be related to genetic traits, such as smaller intron sizes. To this end, we first identified the coordinates of all exons and introns for each gene model in the genome of *P. orbignyanus* and other twenty-six fish species. Using these data, we determined the size of all exons and introns and inferred the size of the genes (exons + introns sizes) (Fig. 6). The estimated and measured C-values for these species show a partial correlation with the size of genes and introns, but not with exon size (Supplementary Fig. S1). In this sense, the total gene size is reduced in *P. orbignyanus* compared to each species analyzed here (Fig. 6A). Similarly, G. aculeatus (Perciformes) and T. nigroviridis (Tetraodontiformes), species with small genomes, also showed a trend toward smaller gene size, which is also confirmed in the percentile analysis of gene size distribution (Supplementary Fig. S2 and Supplementary Table S3). Note, however, that this is not a general trend, as other species with small genomes (B. splendens or C. semilaevis) did not show a general reduction in gene size (Fig. 6A). Also, the distribution of gene size in species with larger genomes did not necessarily show a trend toward longer genes, as is the case of Cypriniformes genomes (Fig. 6A). Note that the total number of genes in *P. orbignyanus* was comparable to that of other Pleuronectiformes species (Fig. 6B and Supplementary Tables S3), which falls within the range of 20–30 thousand genes observed in most species. Interestingly, the genome of T. nigroviridis had the lowest number of genes (14,075). Conversely, other species with large genomes typically had higher numbers of genes, such as Cypriniformes (~ 32–52 thousand genes) and, especially, Salmoniformes (~ 51–180 thousand genes). These results suggest that major genomic rearrangements such as duplication/deletion within coding gene regions, may also be a feature that explains genome size in teleost fishes.

### Genetic features (exons and introns) number and size

**Fig. 7:**
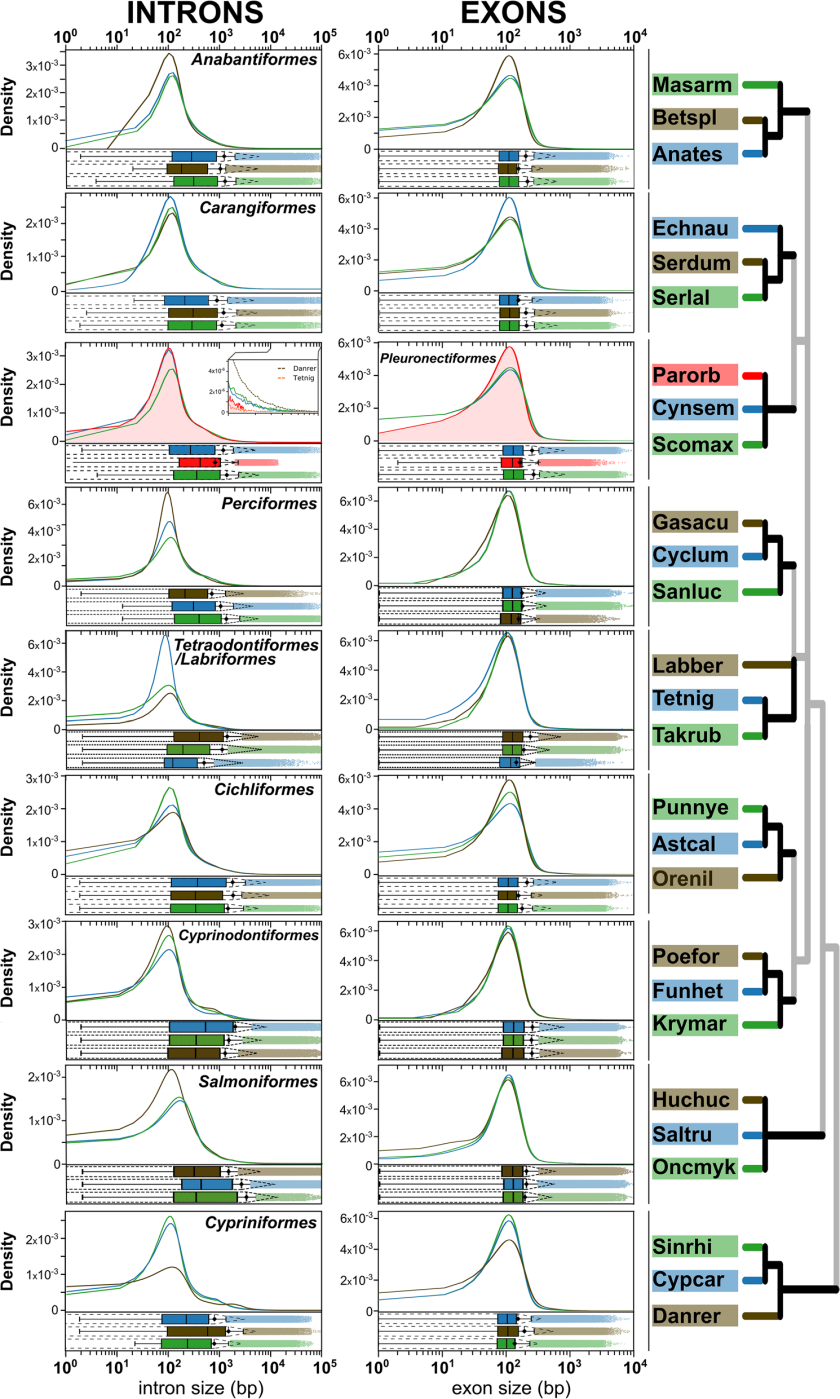
Size distribution of whole genome set of introns (left) and exons (right) for 27 species in 10 fish orders. In x-axis the size in bp is shown (log10 scale). KDE plots represent probability distribution of sizes (y-axis). Below each KDE plot, the boxplots represent the median (line) and quartiles 25 and 75%, whereas whiskers represent the upper and lower bounds. Mean (◆) ± s.d (dashed lines) are shown. Outliers set is shown with dots. Six letter code for the species analyzed as described in Fig. 1 (Onkmyk: *Oncorhynchus mykiss*)

Next, we examined the introns and exons size distribution in fish genomes at the whole genome level. In general, we found that the differences between average and median are smaller for exon sizes when than for intron sizes, suggesting a more compact distribution of introns. It should also be noted that tails towards higher feature size were larger for introns, suggesting that variability in size leads to larger introns rather than larger exons (Fig. 7).

We found no major differences in the distribution of exon sizes between species. The median distribution ranged from 119 bp (*T. nigroviridis*) to 132 bp (*F. heteroclitus*), while interquantile values 75th-25th difference (IQR) span from 84 to 105 bp (*S. rhinocerous* and *P. formosa*, respectively). Among Pleuronectiformes, *P. orbignyanus* (median size value = 127 bp, IQR = 95 bp) had a smaller number of large exons, with the shift occurring more evidently at the 80th quantile (Fig. 7, Supplementary Fig. S3 and Supplementary Table S4). This shift in large exon size was also observed in species with small genome size, such as *T. nigroviridis* and *G. aculeatus*. This had an effect on protein size, as we observed a shift toward smaller predicted protein size in Black flounder compared with other fish species, including Pleuronectiformes, *T. nigroviridis* and *D. rerio* (Supplementary Fig. S4).

Larger differences between species were observed in intron sizes (Fig. 7, Supplementary Fig. S3 and Supplementary Table S5). Median size values ranged from 121 bp (*T. nigroviridis*) to 1139 bp (*D. rerio*), and IQR values were also highly variable. However, in *P. orbignyanus,* we did not detect a significant reduction in intron size at the genomic level. The largest difference in Pleuronectiformes was observed for large introns, with very large introns (> 10,000 bp) being extremely rare in *P. orbignyanus* (Fig. 7, see insert). The 99th quantile for this species is 7311 bp, whereas in other Pleuronectiformes it is 16,966 bp (*C. semilaevis*) and 19,470 bp (*S. maximus*) (Supplementary Fig. S3 and Supplementary Table S5). The 99th quantile in other orders was highly variable: in *T. nigroviridis*, the value of the 99th quantile was 6002 bp and increasing up to 40,237 bp in *D. rerio*. These observations are consistent with the intron size distributions for these two species, where there is a shift towards lower sizes in *T. nigroviridis*, and a shift towards larger intron sizes in *D. rerio*, including an evident shoulder at ~ 1000 bp (Fig. 7). Finally, *P. orbignyanus* genes had few small introns, starting at the 1th quantile (73 bp). In summary, intron size variability may contribute to explain genome shrinkage (as in *T. nigroviridis*) or expansion (as in *D. rerio*). To some extent, our results suggest that this may have an impact to explain *P. orbignyanus* genome shrinkage compared to or other Pleuronectiformes. In this sense, the lower amount of very large and small intron contributes to reduce the overall coding genes size in *P. orbignyanus*.

## Discussion

Teleosts comprise the largest group of vertebrates, encompassing over 35,000 described species [68], each exhibiting a remarkable diversity of forms and functions. Approximately two decades ago, the first complete genome of a teleost, the Japanese pufferfish (*Takifugu rubripes*), was sequenced [31]. Subsequently, the number of fish species with sequenced genomes has grown continuously, primarily owing to advances in sequencing technologies and assembly algorithms [69]. According to NCBI, only about 3% (1071) of all fish species have had their genome sequenced as of August 2023. However, these figures are quickly becoming outdated due to the large-scale sequencing initiatives focused on fish species genomes. One such initiative is the 10,000 Fish Genomes Project (Fish10K) [70] which is dedicated to sequencing and obtaining reference genomes from representative fish species. Recently, FISH10K has adopted long-read sequencing and Hi-C technology to enhance the quality of reference genomes, aiming to cover at least one representative species from each of the fish families. The data collected from Fish10K, in conjunction with genome sequences from other research laboratories (as the work presented in this study), will play a pivotal role in advancing breeding programs, promote sustainable aquaculture, and enabling genome editing and genomic selection [71].

Employing next-generation sequencing (NGS) technologies, we successfully sequenced, assembled, and annotated the genome of non-model species, the Black flounder. The genome annotation identified 25,231 protein-coding genes, surpassing the count of 21,787 protein-coding genes reported for the Japanese flounder [22]. The estimated genome size of Black flounder is approximately 538 Mbp, which aligns closely with the genome sizes of other flatfish species, such as the Japanese flounder (534 Mb) [22], Turbot (568 Mb) [72], Spotted halibut (556 Mb) [24]. It is around 15% larger than the genome of the tongue sole genome (477 Mb) [19] and approximately 11% smaller compared to the Senegalese sole genome (~ 612 Mb) [73]. Furthermore, eight other flatfish species exhibit genomes sizes spanning from 399.64 Mb in Ocellated flounder to 643.91 Mb in Japanese flounder [1].

To determine the genome size conclusively, it is advisable to employ multiple complementary methods to assessing genome quality [74]. In this study, we evaluated the N50 sizes of contigs and scaffolds, as well as genome completeness using KOG and BUSCO. The quantitative BUSCO analysis revealed a high percentage of conserved orthologs in turbot [60]. Notably, the combined assemblies yielded high BUSCO values (94.7%) in Actinopterygii, serving as a compensation for the absence of long reads. It is evident that future transcriptome and long-reads sequencing studies will be imperative to provide a comprehensive, quantitative evaluation of the level of completeness achieved.

The comparative analysis of homologous genes reveals that 1234 putative orthologous groups are exclusive for the three different flatfish families (Scophthalmidae, Cynoglossidae, and Paralichthyidae) (Fig. 3). Within clusters, several semantically similar GO terms correspond to features that are crucial for adaptation to a benthic lifestyle (Supplementary Table S2). It is noteworthy that a recent comparative genome analysis has explored the origins of flatfish body structure and other significant features from an evolutionary perspective, utilizing the genomes of eight new species representing fourteen families of Pleuronectiformes [1]. This study identified certain gene clusters shared by Pleuronectiformes associated with these features. However, more comprehensive comparative analyses that include the South American black flounder can be conducted once the annotated genomes of these species become publicly available.

In this study, we conducted a comparative analysis of 27 flatfish species across 10 different orders to investigate the reasons behind the relatively small genome size observed in the Black flounder. Genome sizes in fish exhibit a wide range, from very compact genomes, such as in *Tetraodon nigroviridis* (~ 350 Mb) [75], to considerably larger genomes, exemplified by *Salmo salar* (2967 Mb) [25]. As expected, our comparative analysis confirmed that the Black flounder has one of the smallest genomes among the species examined, which is consistent with the results of previous studies in which flatfish genome sizes were among the smallest of all teleosts [60, 72]. This observation is further supported by the analysis of fish C-values (Figs. 4 and 5). Specifically, the genomes of *B. splendens* (Anabantiformes), *G. aculeatus* (Perciformes, suborder Gasterosteiformes), *C. semilaevis* (Pleuronectiformes), *T. rubripes,* and *T. nigroviridis* (Tetraodontiformes) in the selected dataset belong to groups with typically small genome size, according to the average C-value (Fig. 4). Moreover, numerous C-values within the Pleuronectiformes family fall within the 10th percentile of the distribution, particularly in the families Paralichthyidae (which includes the Black flounder), Rhombosoleidae and Pleuronectidae. The large genome size observed in salmonids may be attributed to a specific whole-gene duplication event (known as 4R) in this lineage [32]. Although genome size diversity in teleosts is likely connected to the remarkable diversity in morphology, ecology, and behavior within this group [76], the underlying evolutionary forces driving this diversity remain unclear. The variation in genome size remains a subject of debate among evolutionary biologists. In this context, general DNA components implicated include REs, introns, and coding sequences, although their importance varies among plants, fungi, and animals [77]. This complexity poses challenges in developing a comprehensive theory to account such variations. The ongoing debate regarding whether the general mechanisms controlling genome size involve selection or drift adds further controversy [78]. Collectively, these factors limit our ability to draw precise conclusions regarding adaptive advantages linked to the observed genome shrinkage in Pleuronectiformes.

Larger genomes usually contain more extensive intergenic regions with regulatory elements that provide organisms with adaptability in various environments [79]. As a result, the benefits of having a smaller genome in an organism are unclear. A smaller genome might hinder the generation of new gene regulatory events, thereby limiting its adaptability to varying conditions.

Genome size is not only affected by the teleost-specific rounds of wholegenome duplications (3R and 4R) [80]. Other genomic structures have also been linked to variations in genome size. It is well-established, that alterations in the proportion of repetitive elements (REs) can lead changes in genome size [25, 81]. In addition to REs, variations in the numbers of exons and introns may provide further insight into changes in genome size in teleost fish [82].

Many of these REs originates from specific sequences that replicate and move within the genome, the TEs [83]. Therefore, the Black flounder aligns with the trend of compact genomes observed in other flatfish genomes, with approximately 5% TEs, slightly more than the 3% observed in pufferfish, another species with an extremely small genome [31]. Hence, the findings of this study are consistent with the widely accepted understanding that genome size variation eukaryotic species is linked to the amount of repetitive DNA [84], a phenomenon also observed in teleost fishes [85]. The abundance of TEs varies in accordance with both genome size and position of species within the fish tree of life [86]. In this research, we compared the proportion of various REs (Fig. 1), including TEs, LINEs, SINEs and LTRs, across 27 fish species. In Black flounder and the Pleuronectiformes orders in general, the overall proportion of REs was relatively low, which is likely one of the contributing factors to the small size of the Black flounder genome. Interestingly, our study (Fig. 2) unveiled an exceptional abundance of TEs in zebrafish. However, it is particularly surprising that despite the high number of TEs, the zebrafish genome itself is not exceptionally large (Fig. 2). Yet, this does not offer a clear explanation for the lack of direct correlation between the proportion of TEs and the total genome size in this particular species, warranting further research to shed light to this intriguing phenomenon.

Variations in the frequency and position of transposons within the genome can have a major impact on the physiology of species. TEs are well-documented to play an important role in shaping genome structure and stability, thereby impacting various evolutionary and ecological processes, including the generation of biodiversity, stress responses, adaptation, and speciation [87]. For instance, a change in the position of a TE disrupted a specific single gene, resulting in multiple phenotypes changes, ranging from alterations in color and reduced growth performance to increased locomotion [88].

As previously mentioned, another factor capable of influencing genome size is the number and size of exons and introns. In this regard, we determined the coordinates of all coding genes in the genomes of twenty-seven fish species, including *P. orbignyanus*. The results revealed that *P. orbignyanus* exhibits smaller gene sizes when compared to all other fish species examined, even those belonging to groups with small genomes, such as the Tetraodontiformes [35] and certain Perciformes [89]. Flow cytometric analyses indicated that in four pufferfish species within the Tetraodontidae family, genome size ranges from 0.38 and 0.82 pg, whereas the sister family Diodontidae possesses larger genome sizes (0.8–1 pg), likely due to DNA loss during the evolution divergence of the families [90]. Our findings are consistent with this pattern, as we have demonstrated that Pleuronectiformes, in general, exhibit small genome size ranging from 0.3 to 1.1 pg, making them among the smallest of all teleost fishes. Given that *T. nigroviridis* also demonstrates a tendency toward smaller gene sizes, this could represent one of the potential explanations for genome shrinkage.

In eukaryotes, intronic DNA represents a significant component of genes and genomes, playing a key role in gene regulation, and intron size holds significance from an evolutionary standpoint [30]. In teleosts, there exists a close relationship between genome size and intron size, with intron size mirroring genome size [82]. To further investigate the factors contributing to the small genome size of the Black flounder, we examined the distribution of total genome size concerning introns and exons across 27 species belonging to 10 fish orders. Our analyses confirmed the expected pattern where the mean and median exon sizes were smaller in comparison to intron sizes, suggesting a more compact distribution, which is consistent with the observations in other Pleuronectiformes species [2]. However, we observed a distinct distribution of the intron size in the genome of the Black flounder, particularly marked by a decrease in the number of very large and small introns (Fig. 7 and Supplementary Fig. 4). Indeed, the intron size distribution in other species with small genomes, such as *T. nigroviridis* and *T. rubripes*, also reflects smaller intron sizes, suggesting that this may be a mechanism contributing to genome size reduction in these species. However, the scarcity of small and very long introns in *P. orbignyanus* could account for the observed reduction in gene size within this species. Concerning exon size, our findings were consistent with previous studies, as we did not observe any significant differences in exon size distribution among species [91]. Based on these results, we can infer that intron size may play a role in gene size and, consequently, genome shrinkage in the Black flounder, particularly in relation to the reduced contents of very large and small introns. Further comparative analysis involving genomes annotated at chromosome level within Pleuronectiformes and other fish species with notably small or large genomes (such as *T. nigroviridis* and salmon genomes) may shed light on other mechanisms at play, including the size of intergenic regions.

Alterations in gene structure, such as the reduction in intron size, can significantly impact alternative splicing (AS) of genes. AS is an essential mechanism that plays a key role in cellular differentiation and organism development [92]. In teleosts, lower AS frequencies have been observed in highly duplicated genomes (*e.g*., zebrafish) and large occurrences in compact genomes (*e.g*., pufferfish). These inverse correlations between AS frequency and genome size appear to be the same across fish species [93]. This study initiates a new research direction, questioning whether smaller introns may influence the AS mechanism [94–96]. The increasing abundance of data from transcriptome analysis and fish genomes proves invaluable for conducting comparative genome studies and investigating the potential correlation between alternative splicing and genome size through RNA-seq analysis. The investigations of alternative splicing hold great significance as it offers insights into the intricate and dynamic processes of gene regulation. Not only does it deepen our understanding of how genes are finely tuned to perform diverse functions, but it also sheds light on the complexity of gene expression, providing valuable insights into the molecular basis of various biological processes [92].

Over the past decade, numerous genomic, proteomic, and metabolomic studies have been dedicated to characterizing aspects of reproduction, development, nutrition, immunity, and toxicology in flatfish [97, 98]. Flatfish genomics is important for studying the management of wild fish populations, improving fish conservation, and increasing productivity in aquaculture [16]. In the recent years, long-read sequencing technologies have been applied to several pleuronectiform species, facilitating the assembly of chromosomes [1, 21, 23, 24, 99]. High-quality reference genomes are important for studying evolutionary variation in fish genome structure and organization [100]. Furthermore, *P. orbignyanus* is categorized as ‘data deficient’ in the IUCN Red List of Threatened Species [101], making genetic variation at the genomic level of this species a potential starting point for future studies into population genetic structure within this specie. This technology will enable us to conduct new comparative and in-depth analyzes. For instance, an important outstanding question is whether there exists a general sex-determining gene (*locus*) in fish and particularly in flatfish.

Sex determination is the genetic or environmental process by which the sex (gender, male or female) of an individual is established in a simple binary fate decision. Traditionally, in fish, two forms of sex determination were described: Environmental (ESD) and Genotypic (GSD) sex determination. These two forms were once considered to be mutually exclusive, however, it has been shown that they can coexist in some conditions [102]. The knowledge of sex determination in this group has not only importance in the advance of basic physiology but also in the control of sex ratios in fish farming.

In this sense, a recent study by Ferchaud et al. [103] propose the SRY-Box Transcription Factor 2 (*Sox2*) as a potential candidate not only in Greenland halibut but also in other flatfishes. Other gene candidates include follicle stimulating hormone receptor (*fshr*) in Senegalese sole [104], bone morpho-genetic protein receptor type1B (*bmpr1ba*) in *Hippoglossus stenolepi* [99], Forkhead box L2 (*Foxl2)* and Doublesex and mab-3 related transcription factor 1 (*dmrt1)* in Japanese flounder (*Paralichthys olivaceus*) [105], and Gonadal soma-derived factor (*gsdf*) in Atlantic Halibut (*Hippoglossus hippoglossus*) [106, 107]. Furthermore, another recent study conducted in the Japanese flounder, utilizing *amhy*-mutant flounders generated thorough the CRISPR-Cas9 system technology, demonstrated the crucial role of *amhy* for testicular formation in this species [108]. In this study, we thoroughly explored and identified all genes associated with gene markers in flatfish, as mentioned earlier (see Table 4). Given that all genes linked to sex determination in flatfish have been found in the genome of Black flounder, we propose that the first attempt to study the mechanism of sex determination in this species should concentrate on exploring the roles of *dmrt1* and *amhy*, since both genes have been already studied in Japanese flounder, a species of the same genus.

**Table 4:** Summary of sex determination (SD) genes described in flatfish

Species (Common names)	Gene name (Abbreviation)	Physiological actions in reproduction fish	References
*Reinhardtius hippoglossoides* (Greenland Halibut)	SRY-Box Transcription Factor 2 (*sox2)*	role in sex determination and differentiatio*n*	Ferchaud et al., 2021 [103]
*Solea senegalensis* (Senegalese sole)	Follicle stimulating hormone receptor (*fshr)*	*r*ole in folliculogenesis	De la Herrán et al., 2023 [104]
*Hippoglossus stenolepis* (Pacific Halibut)	bone morpho—genetic protein receptor type-1B (*bmpr1ba*)	potential candidate for master sex‐determining	Jasonowicz et al., 2021 [99]
*Paralichthys olivaceus* (Japanese flounder)	Forkhead box L2 (*foxl2*)	role in ovarian differentiation	Shu et al., 2021 [105]
	A male-specific duplication of anti-Müllerian hormone (*amhy*)	role in sex determination and differentiation	Hattori et al., 2021 [108]
	Doublesex and mab-3 related transcription factor 1 (*dmrt1)*	roles in sex determination and neural development	Shu et al., 2021 [105]
*Hippoglossus hippoglossus* (Atlantic Halibut)	Gonadal soma-derived factor (*gsdf*)	role in testicular differentiation	Palaiokostas et al., 2013; Einfeldt et al., 2021 [107, 106]

In summary, in this study we generated a genome assembly of Black flounder (*Paralichthys orbignyanus*). Our findings reveal a reduced genome size and lower frequency of REs in the flounder. We have established that phenomenon is correlated with a reduction in the size of gene *loci*, primarily attributed to a decrease in the number of very large and small introns. Based on the analyzed features, we have reached the conclusion that the primary factors potentially responsible for the reduction in the flounder genome include (i) the low frequency of repetitive elements, (ii) the overall reduced gene size, which is likely associated primarily with (iii) the decreased number of both very large and small introns, with potential implications for AS. The last two components (ii and iii) exhibit lower values compared to other species with similar Cvalues, suggesting that this may represent a novel strategy for genome reduction.

Potential future research directions that could emerge from these findings involve comparative genomics with other teleost species, contributing to a better understanding of fundamental biological questions, such as shedding light on the genome size strategies in teleost fishes. Furthermore, our contribution can support future efforts aimed at developing sustainable aquaculture strategies for the Black flounder in South America.

### Supplementary Information

**Supplementary material 1.**

**Supplementary material 2.**

**Supplementary material 3.**

**Supplementary material 4.**

**Supplementary material 5.**

**Supplementary material 6.**

## Data Availability

This sequencing data has been deposited at the National Center for Biotechnology Information (NCBI) where the Bioproject Accession ID is PRJEB36690.
