# Supplementary material for: Genome sequencing and analysis of black flounder (Paralichthys orbignyanus) reveals new insights into Pleuronectiformes genomic size and structure
Source: BMC Genomics. 2024 Mar 20;25:297. doi: 10.1186/s12864-024-10081-z (PMC10956332; doi:10.1186/s12864-024-10081-z)
Supplement: Supplementary file 1 — Supplementary material 1. [file 12864_2024_10081_MOESM1_ESM.pdf]

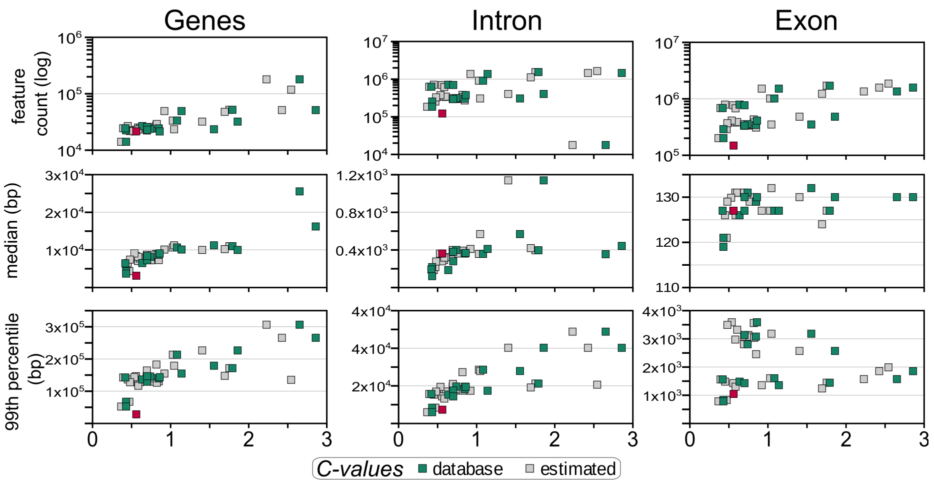


**Supplementary Fig S1**

Correlation plots for C-values (as per Animal Genome size database or estimated from sequenced genome, when available, in x-axis) vs feature count (top, log scale), feature median size (center, bp) and feature 99$^{th}$ percentile size (bottom, bp). Features analyzed are Genes, Intron and Exons (in columns from left to right). Data corresponding to *P. orbignyanus* highlighted in red.

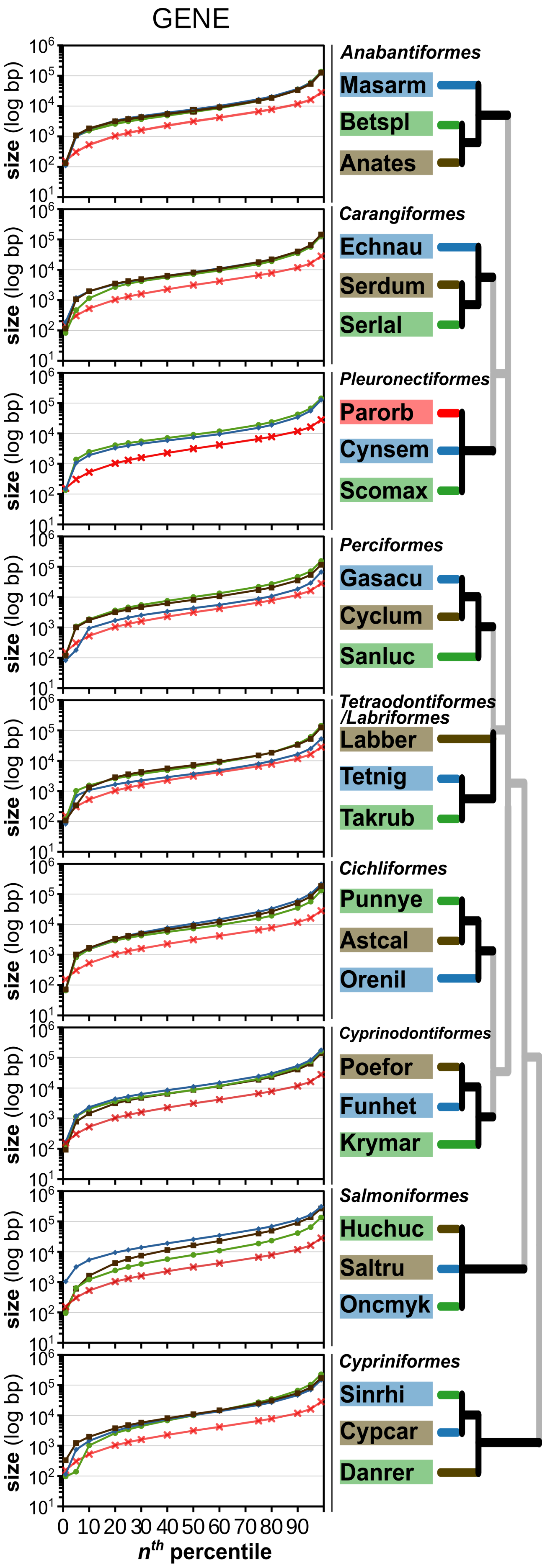


**Supplementary Fig S2**

**Gene size percentile distribution.**

For each species, gene size distribution $n^{th}$ percentiles ($n$ 1, 5, 10, 20, 25, 30, 40, 50, 60, 75, 80, 90, 95 and 99) were computed (shown in y-axis size, bp in log scale). *P. orbignyanus* data (red) is shown in all plots for comparison. Six letter code for the species analyzed as described in **Fig. 1** (Onkmyk: *Oncorhynchus mykiss*).

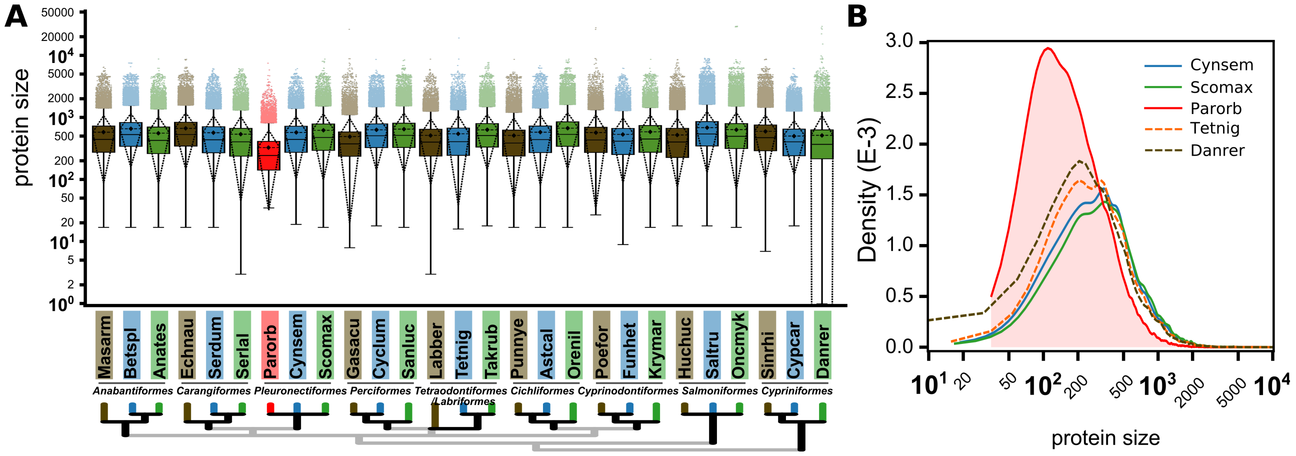


**Supplementary Fig S3**

**Black flounder predicted protein size distribution.**

**A.** Complete fish proteome size distribution. Horizontal bar shows median, whereas dotted lines represent standard deviation, and diamonds represent mean value. Outliers are shown as filled circles. **B.** Kernel density estimation plot of protein size distribution in Pleuronectiformes (Cynsem, Scomax and Parorb), plus representatives from Tetraodontiformes (Tetnig) and Cypriniformes (Danrer). Six letter code for the species analyzed as described in **Fig. 1** (Onkmyk: *Oncorhynchus mykiss*).

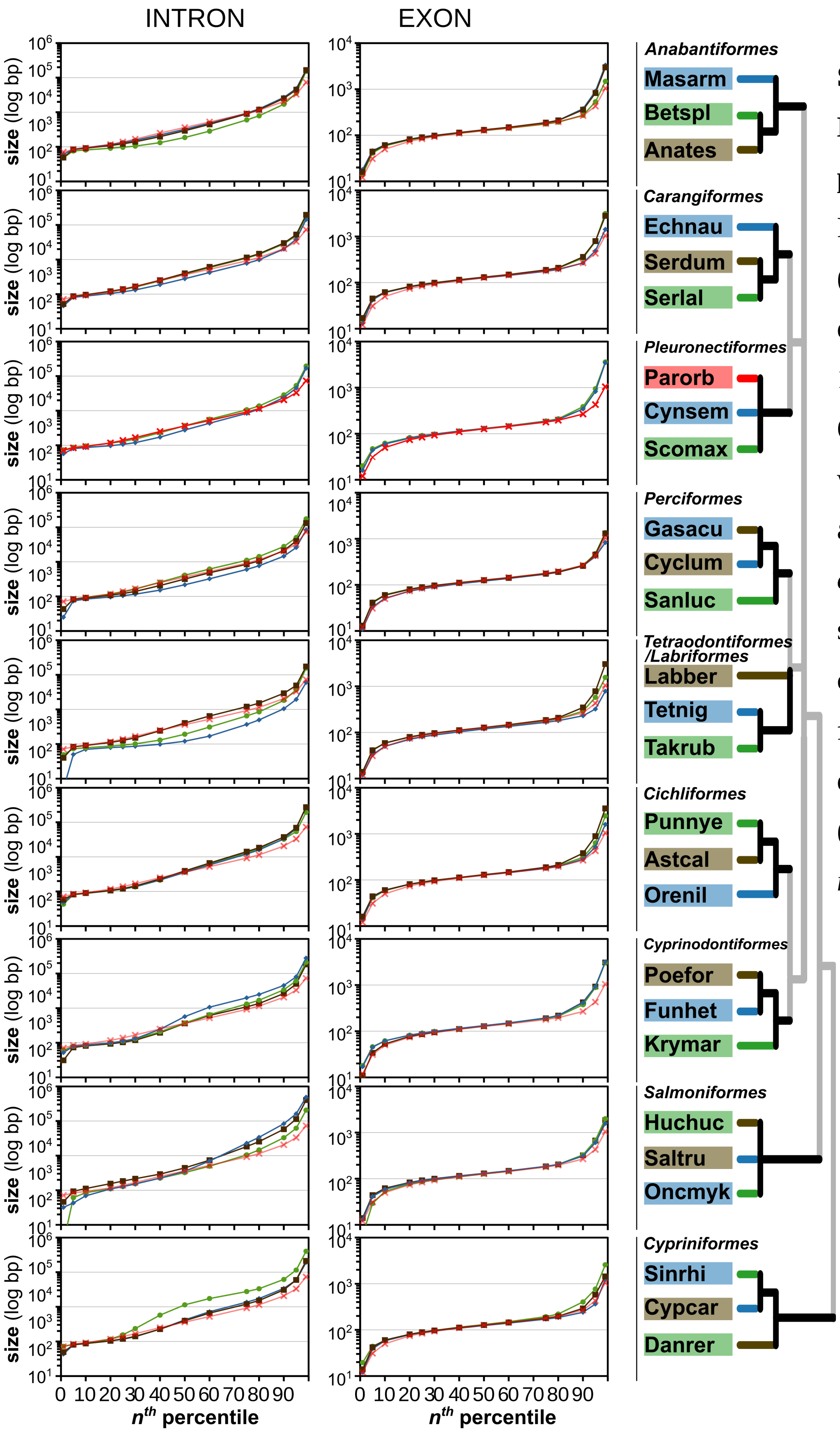


**Supplementary Fig S4**

**Exons and introns size percentile distribution.**

For each species, intron (left) and exon (right) size distribution $n^{th}$ percentiles ($n$ 1, 5, 10, 20, 25, 30, 40, 50, 60, 75, 80, 90, 95 and 99) were computed (shown in y-axis size, bp in log scale). *P. orbignyanus* data (red) is shown in all plots for comparison. Six letter code for the species analyzed as described in **Fig. 1** (Onkmyk: *Oncorhynchus mykiss*).
